# Breaking barriers: A study protocol on unveiling gender, racial and other intersectional dynamics in post-secondary institutions and identifying solutions for advancing primary care and public health research

**DOI:** 10.1371/journal.pone.0344467

**Published:** 2026-03-17

**Authors:** Monica Aggarwal, Sabrina T. Wong, Andrea C. Tricco, Tanvir C. Turin, Aisha Lofters, Gina Agarwal, Barnini Bhattacharyya, Ivy Lynn Bourgeault

**Affiliations:** 1 Dalla Lana School of Public Health, University of Toronto, Toronto, Ontario, Canada; 2 Department of Family and Community Medicine, University of Toronto, Toronto, Ontario, Canada; 3 Centre of Health Services and Policy Research, Vancouver, British Columbia, Canada; 4 School of Nursing, University of British Columbia, Vancouver, British Columbia, Canada; 5 Li Ka Shing Knowledge Institute, St. Michael’s Hospital, Unity Health, Toronto, Ontario, Canada; 6 Department of Family Medicine, Cumming School of Medicine, University of Calgary, Alberta, Canada; 7 Women’s College Hospital Research and Innovation Institute, Toronto, Ontario, Canada; 8 Department of Family Medicine, McMaster University, Hamilton, Ontario, Canada; 9 Ivey Business School, Western University, London, Ontario, Canada; 10 School of Sociological and Anthropological Studies, University of Ottawa, Ottawa, Ontario, Canada; PLOS: Public Library of Science, UNITED KINGDOM OF GREAT BRITAIN AND NORTHERN IRELAND

## Abstract

**Background and objectives:**

This study protocol employs critical race and intersectionality theories to investigate barriers faced by racialized women at various academic career stages within Canadian primary care (PC) and public health (PH). The objectives are to identify faculty characteristics, examine intersectional barriers, and recommend equity-focused, inclusive strategies and policies.

**Research design and methods:**

The study adopts a sequential mixed-methods approach. A quantitative survey and/or existing datasets will be used to collect demographic data on PC and PH academic position holders in Ontario and British Columbia, Canada. Data will also examine experiences of workplace discrimination; equity, diversity and inclusion (EDI) resource use; and departmental satisfaction. Subsequently, we will conduct interviews with researchers and leaders who are responsible for hiring and involved in or addressing matters related to EDI. Inductive and deductive approaches will be used to analyze the data in accordance with theoretical frameworks to deepen insights into equity and inclusion in academia.

**Results:**

The quantitative phase will profile PC and PH academic position holders, highlighting disparities in positions and leadership roles. The qualitative study will explore intersectional challenges faced by racialized women academic position holders during career progression. Preliminary findings will inform effective equity-promoting strategies.

**Discussion and implications:**

This study aims to contribute to the existing body of knowledge on gender and racial inequities in academia by uncovering systemic identity-based disparities in the careers of PC and PH researchers in Canada. The findings will inform the development of targeted interventions to promote equitable hiring, faculty support, and leadership advancement, enhancing diversity and productivity through an inclusive and equitable academic environment. Findings will be shared via publications, policy briefs, workshops, and online platforms to engage academics, advocacy groups, funders and policymakers in promoting equity and driving institutional change in PC and PH research.

## Introduction

Research is vital for advancing primary care (PC) and public health (PH), particularly amid the ongoing PC crisis in Canada [1,2]. Effective research informs policymaking and meets community needs [3]. A diverse academic workforce is essential for fostering innovation [4], critical dialogue [5], research productivity [6], and producing rigorous research that benefits patients and all members of society [7]. However, research shows that individual and organizational systemic bias on the basis of gender and race hinders research productivity (often measured by publication count, citation count, and h-index) [8] across health fields [9] and career stages [10,11], which can lead to burnout [12,13] and workforce attrition [14].

Women and racialized academic position holders have lower publication rates compared to white men [15,16]. A recent systematic review showed that women in academic medicine had lower publication productivity than men across all career stages and all specialties [17]. Even with equal collaboration with men in medicine and health sciences, first-authored articles by women receive fewer citations [18]. Globally, women, specifically racialized women, in health specialties also face challenges with securing funding from granting agencies [19–24], further worsening racial inequities in early-career researchers and negatively impacting career advancement [25]. Discriminatory practices and biases in the academic workplace impede the research productivity of women and racialized academic position holders [26–28], from entry-level to faculty and leadership roles.

Research consistently shows that women, especially racialized women, face challenges with being offered and entering faculty roles in medicine. More male faculty compared to women faculty are hired across medical specialties except obstetrics and gynecology [17]. A recent study showed significant inequalities in United States faculty hiring and retention, with limited progress toward long-term gender parity in hiring practices [29]. Furthermore, racialized women academic position holders are persistently underrepresented in medicine, science and social science fields [30–39]. When they are hired, they are often in non-tenured contractual positions, which further limits their advancement, salary, and leadership power [40]. Women of colour may hesitate to speak out against the injustices they face personally due to concerns about being perceived as disruptive [40] and employer retaliation [41].

As faculty, women have lower research productivity. This has been attributed to inadequate institutional support, especially for racialized women. This includes a need for more support for financial resources, mentorship, social capital and fair performance expectations. Parenthood further diminishes the research productivity of women, with mothers producing significantly fewer papers than fathers after childbirth [42]. The COVID-19 pandemic exacerbated inequities for women, leading to reduced publications and grants due to greater caregiving responsibilities [43]. A study involving Science, Technology, Engineering, and Mathematics faculty [43] found that 43% of women, compared to 23% of men, exited full-time employment following the birth of their first child [43]. Inadequate parental leave policies and childcare facilities further accentuate these challenges [44]. For example, grantees and post-doctoral fellows are not eligible for parental leave [45], leading to expectations that they continue their work during maternity leave.

Workplace cultures also serve as barriers to research productivity. Studies indicate that workplace culture often excludes women, particularly racialized women, from research networks and networking events [46–48]. This exclusion is due to discriminatory practices rooted in biases and stereotypes, which hinder the research productivity of women. Toxic work environments are prevalent in medical specialties, where harassment, bullying and microaggressions directed towards women and racialized women are common issues [30,49,50]. In Canada, research shows that racialized women researchers have been the targets of harassment from colleagues and leaders in the workplace. Employers foster these cultures by discouraging racialized women from speaking up by instilling fear of retaliation [51–53] or hosting events at times or locations when women are unable to attend [54], allowing microaggressions and harassment to continue in the workplace. The literature also suggests women faculty spend more time on service and teaching compared to their male counterparts [55], with racialized women academic position holders assigned the most undesirable tasks [56]. Biases that racialized women are less credible and lacking in skills and intellect [57] can result in devaluing their scholarship through lack of recognition or attributing credit to colleagues [58].

During career advancement, women are also underrepresented in leadership positions in academic medicine [59,60], including dentistry [61], emergency medicine [62], gastroenterology [63–65], cardiology [66,67], general surgery [68], pediatrics and geriatrics [60]. In 2019, women in the United States comprised 19% of the total number of chairs in medical schools. Black, Indigenous, and People of Color and Underrepresented Minority women constituted only 24% and 15% of female chairs and 5% and 3% of all chairs in medical schools, respectively [69]. Barriers in medicine and biological sciences fields include implicit biases [61,70–72], limited mentorship opportunities [73–77], scarcity of women role models [78,79], and discrimination and harassment due to racism and ethnicity [26,80,81].

While there is existing research on sex, gender and racial disparities in academia (science and medical specialty disciplines), our comprehensive review of the literature indicates there are no studies that examine the role of intersecting social identities in PC and PH research during different career stages – at entry, as faculty and in leadership. In addition, there is a lack of knowledge of how the operational dynamics of PC and PH departments and institutions support or hinder equity, diversity and inclusion (EDI) efforts. Departmental dynamics (contexts) refer to the culture, policies, and power structures within specific academic departments, directly affecting faculty members’ day-to-day interactions, access to resources, and support within their immediate work environment [82]. Institutional dynamics, on the other hand, encompass the broader culture, policies, and structural frameworks of the entire university or organization [83], such as hiring processes [84].

### Key objectives of this study include

Objective 1: Identifying demographic characteristics of academic and leadership position holders in PC and PH departments.

Objective 2: Exploring intersectional experiences of racialized women academic position holders to highlight the systemic, institutional and departmental barriers faced by researchers at entry, as faculty and in leadership roles. This includes examining experiences of discrimination and microaggressions, as well as how multiple dimensions of marginalization (e.g., sexual identity, disability, age, immigration status, etc.) intersect with race and gender across career stages. This exploration will capture the nuances of how different identities and backgrounds influence faculty members’ experiences within PC and PH.

Objective 3: Recommending actionable strategies and policies that can be implemented at the system, institutional and departmental levels within PC and PH research environments to foster an inclusive culture that amplifies diverse voices and ensures equitable recognition of contributions across the academic landscape.

### Theoretical orientation

The proposed study will combine Critical Race Theory (CRT) [84,85] and Intersectionality Theory [86,87]. CRT contends that racism is not merely based on individual acts of prejudice but deeply ingrained in societal structures and institutions, perpetuating systemic inequalities [85]. The theory underscores the importance of understanding how policies and institutions contribute to and maintain racial disparities, emphasizing the importance of understanding the lived experiences of marginalized groups and their historical contexts [85]. While the primary analytic focus remains on racialized women, the study explicitly attends to other intersecting dimensions of marginalization, including sexual identity, disability, age, immigration status, and related identity-based factors. Intersectionality Theory complements CRT by exploring how multiple social identities intersect to create unique experiences of oppression and privilege [88–90]. Intersectionality theory aligns with feminist theory in its shared focus on power structures, social inequality, and the lived experiences of marginalized individuals, particularly women. Both theories aim to deconstruct traditional narratives that oversimplify or ignore the experiences of those who are marginalized and emphasize understanding experiences through a more holistic, inclusive lens [91,92]. Both feminist and intersectionality theories are concerned with understanding how systems of power (patriarchy, racism, capitalism) perpetuate inequality. Intersectionality adds depth by analyzing how these systems overlap and intersect, creating unique experiences of oppression for women with multiple marginalized identities [91].

CRT and Intersectionality Theories have been used across disciplines within academia [93–95]. We will combine these theories as a conceptual framework to understand the experiences of women researchers and leaders with various identities and to identify solutions for structural changes within academic institutions to address systemic racism and address disparities in funding, scholarships, and access to educational resources [87,88]. In this study, we differentiate between “female” and “male” as biological categories, referring to individuals’ physiological attributes, and “women” and “men” as social and cultural identities that encompass the roles, behaviours, and experiences shaped by societal norms [96]. We recognize that while “female” and “male” denote biological sex, “women” and “men” reflect the complex interplay of gender identity, socialization, and cultural expectations.

## Methods

### Study design: Mixed methods study

The study will employ a solutions-oriented [97], sequential mixed-methods design [98] to explore the intersectional barriers faced by female academic position holders, particularly racialized women, in PC and PH research in Canada. First, a quantitative study will collect and analyze demographic data of PC and PH academic and leadership position holders. The survey will be reported using the Strengthening the Reporting of Observational Studies in Epidemiology (STROBE) guidelines, which provide a framework for transparent and comprehensive reporting of observational studies, including those involving surveys, to enhance the quality and reliability of the findings [99]. Second, a qualitative study will be completed using in-depth interviews. The qualitative interviews and surveys will be conducted in accordance with established reporting guidelines, such as the Consolidated Criteria for Reporting Qualitative Research (COREQ) [100]. This design allows for a comprehensive understanding of both the breadth and depth of the challenges faced by women and those who identify as women academic position holders, facilitating the identification of effective strategies to promote equity and inclusion.

### Setting and intervention

The study will be conducted across academic settings conducting PC and PH research, including universities in Ontario (ON) and British Columbia (BC). This will include departments, schools and institutes of family medicine, nursing, public health, health services research, policy and management. By including a diversity of PC disciplines, we aim to understand the impediments to research productivity, regardless of the specific departmental affiliation within institutions. ON and BC were selected as jurisdictions due to the significant proportion of the population identifying as racialized individuals residing [101,102] and the presence of academic institutions with substantial PC and PH research activities.

### Quantitative study (Objective 1)

#### Population sample.

Participants will include academic position holders who are professors in PC and PH academia. We will use the U15 Group of Canadian Universities [103]. This includes the University of Toronto, McMaster University, Western University, Queen’s University, York University, the University of Ottawa, the University of British Columbia, Simon Fraser University, the University of Victoria, Thompson Rivers University, the British Columbia Institute of Technology, and Kwantlen Polytechnic University.

Then, we will collect comprehensive data on the demographic attributes and career profiles of PC and PH researchers. This will include conducting surveys with faculty. Academic position holders interested in participating in the study will document their written consent as part of an electronic survey.

##### Survey.

Demographic data collection will include critical factors that are often unexamined but are essential to understanding the full context of academic position holders’ lives and their impact on career progression. This will involve detailed attention to family and home life, including the presence and age of children, their ages, when they were born (while studying, on the tenure track, etc.). Demographic data collection will be informed by our literature review and include: Tenure Status (tenured and non-tenured roles) [104,105]); FTE status; Gender (gender distribution) [18,106]), Ethnicity or Race (racial or ethnic backgrounds) [107]); Academic Rank (academic positions, including assistant, associate, and full professors [108,109]); Educational Background (educational qualifications and degrees held by researchers, e.g., PhD vs MD vs MD-PhD etc. [110]); Years of Experience (range of experience among faculty members [111]); Institutional Context (exploring institutional characteristics (location, funding sources, and institution size) [110]); Parental Status (dependent children or are caregivers [112,113]), including when children were born during their career [114]); Family Structure (family structures, including single-parent households, dual-career families, and other caregiving arrangements, including elderly parents [112,113]); Marital Status/ Spouse/Partner Career (careers or professional engagements of spouses or partners provides context to researchers’ support systems and potential constraints such as relocation [112]); Salary; Leadership Role (position in organization, salary, leadership training opportunities [115,116]).

An electronic survey will be administered to PC and PH academic position holders to also collect data on their awareness and utilization of EDI resources, such as EDI office initiatives, training programs (i.e., leadership, teaching), mentorship opportunities, and overall satisfaction within the department or institution. The survey will also incorporate validated measures of workplace discrimination [117] and microaggressions [118] to capture faculty experiences of discrimination alongside career trajectories and advancement (S1 File). At the departmental and institutional level, data will be collected on strategic plans and resources. Data collection will begin in March and will be completed in April 2026.

##### Data Analysis.

Descriptive statistical methods will be employed to analyze the quantitative data, focusing on identifying overarching patterns and distributions among PC and PH faculty academic position holders in ON and BC. We will examine characteristics and trends using descriptive statistics to summarize the demographic data of PC and PH faculty [119]. Comparative analyses and subgroup examinations will identify disparities or trends within demographic groups, offering nuanced insights. T-tests, ANOVA, and chi-square tests will be calculated to compare groups based on variables such as age, gender, rank, length of appointment, leadership position, status (tenure versus contract), institution, institutional setting (university, research institutes, hospital) and research focus, where possible. These methods allow for a more granular understanding of how demographic and professional factors influence the characteristics of PC and PH faculty, thereby informing targeted interventions or policies.

### Qualitative study (Objectives 2 and 3)

#### Population sample.

Participants will include women and those who identify as women academic position holders at different career stages (i.e., entry into faculty, as a faculty member and within leadership) and individuals involved in leadership roles and EDI committees and unions representing faculty to understand their experiences with hiring, harassment, complaints, and potential interventions and their effectiveness for an inclusive and diverse workforce. We will employ a maximum variation sampling technique [120] to ensure a diverse representation of participants across different career stages, genders, ethnicities, and academic backgrounds (MD, non-MD, PhD). For leaders, selection will be based on roles (Dean, Chair, Committees, Ombudsmen, Unions) to capture various organizational viewpoints. We will aim for representation from academic institutions in ON and BC to capture institutional diversity.

We will focus on gender rather than biological sex in the recruitment for our study to understanding the nuanced experiences and challenges faced by individuals in academia. By emphasizing gender, we can better examine how societal norms, roles, and expectations influence professional experiences, career progression, and systemic barriers within academia. This focus allows us to explore how gender dynamics impact hiring practices, workplace culture, and perceptions of leadership, particularly for gender minorities.

Using the information gathered in Phase 1, as well as the collaborative partnerships formed with faculty administration, we will invite participants to partake in an interview. Specifically, individuals identified through the Phase 1 data collection process will be sent personalized invitations to participate in the study. The invitation will outline the objectives of the study, emphasize its importance, and highlight the potential benefits of participation as well as its confidentiality, which is important in this situation due to documented fear of retaliation. In addition, we will distribute a flyer to faculty through their administration. Additional reminders will be sent to non-responders to maximize response rates.

We will conduct in-depth semi-structured interviews [121]. Semi-structured interviews allow for capturing participants’ experiences and perceptions of complex topics. Interviews will be adaptable and empathetic to participants’ needs. For example, we will schedule interviews at times and in formats (e.g., in-person, online) that are contextually appropriate and mitigate barriers to participation [122]. Prior to the interview, we will administer a demographic survey to inform purposeful and theoretical sampling [123], and the study findings from the first phase will be shared for comment. Using a maximum variation sampling technique [124], 15 researchers per jurisdiction and 10 leaders with experience in hiring practices or experiences related to EDI will be interviewed one-on-one using a semi-structured interview guide. Drawing from existing literature on gender and racial disparities in academia, the interview guide will focus on factors that impede the research productivity of racialized women academic position holders, including systemic biases and workforce cultures [125–128] (S2 File). Interviews will be conducted by experienced research staff and explore participants’ experiences and perceptions related to: system barriers (i.e., grant competitions, awards, media requests, and recognition); institutional barriers (i.e., supervisor’s role in potential entry into faculty, hiring practices, the role of leadership, harassment and bullying, engagement in collaborations, workplace policies and supports, EDI initiatives); and potential interventions and strategies that can foster an inclusive and diverse workforce for racialized women at all career stages. By aligning the interview guide with insights from the literature, the study ensures that critical topics are covered while also allowing for the exploration of emerging themes and participant-driven narratives. At the same time, to avoid an extractive approach, it’s crucial to view participants not merely as “subjects” but as co-creators of knowledge [129]. As such, we will also ask participants about their own goals and concerns related to the study’s topic.

#### Data Analysis.

Interview data will be audio-recorded and transcribed verbatim by a professional. The transcriptions will then be subjected to an inductive and deductive thematic analysis [130] by allowing themes to emerge from the data while also applying our theoretical frameworks (CRT and intersectionality) to enhance our understanding of the participants’ narratives. Thematic analysis will involve several steps: familiarization with the data, generating initial codes, searching for themes, reviewing themes, defining and naming themes, and producing the final report. An inductive, iterative approach to thematic analysis allows themes to closely align with a participant’s own words and experiences. This method is widely recognized in qualitative research for its flexibility and rigour in uncovering underlying patterns within qualitative data [130]. It facilitates a deep understanding of the participants’ perspectives, which is essential for accurately capturing the complexity of their experiences and viewpoints. Following the initial inductive coding process, we will utilize CRT to explore how systemic racism and power dynamics shape the experiences of participants [85]. Simultaneously, we will apply intersectionality to examine how various identities—such as race, gender, ethnicity socioeconomic status, discipline, clinician versus non-clinician, ethnicity, immigration status, caregiver status—interact to shape participants’ experiences in complex ways [86]. This dual theoretical approach will enable us to identify themes that capture both individual lived experiences and the broader structural factors at play, drawing attention to intersecting inequalities and unique challenges faced by diverse groups within the PC and PH system. Coding and analysis will be an iterative process that will involve the research team frequently returning to the data to ensure no participant voice or narrative is oversimplified [131].

Moreover, our positionality as academic position holders from diverse backgrounds will inform the analysis, promoting reflexivity and minimizing bias in interpreting participants’ narratives. Our team comprises individuals with varying identities, including women, men, and women of color, as well as academic position holders with lived experiences in marginalized communities within academia. Therefore, reflexivity will occur through a structured and ongoing process throughout the research study [132]. We will hold frequent team meetings to discuss our evolving thoughts, experiences, and potential biases as we engage with the data. These discussions will provide a platform for team members to share insights from their positionality and how it might influence the interpretation of participants’ narratives. When analyzing the data, we will actively seek out differing viewpoints within the team. By encouraging open dialogue and critical discussion of the themes and interpretations, we can challenge one another’s perspectives and enrich the analysis with diverse insights. This practice will also encourage individual team members to critically engage with their biases and assumptions, as well as how these may shape their analysis of the data.

Descriptive statistics will also be calculated for variables that describe the characteristics of the interview sample [133]. This includes demographic information such as age, gender, and ethnicity, caregiver status, marital status as well as professional attributes such as rank, institution, and status.

#### Triangulation of the results.

We will combine quantitative survey results, which highlight patterns and trends in demographics, career profiles, and EDI resource utilization, with qualitative insights that delve into personal experiences of systemic, institutional and departmental barriers. This multi-method approach will contextualize quantitative trends within lived experiences, allowing us to explore if and how demographic variables correlate with qualitative themes. For example, if quantitative data show significant differences in research productivity between PC and PH faculty, qualitative data will be examined to understand the contextual factors behind these differences. Likewise, the survey data on resource awareness will be cross-referenced with qualitative insights on the perceived effectiveness of these resources, enabling us to provide a nuanced perspective on EDI initiatives.

Given our team’s diverse gender, racial, and professional identities, we will collaboratively analyze data with an emphasis on reflexivity [134]. Regular team meetings will encourage discussion of biases and assumptions, ensuring diverse perspectives inform the interpretation of the data and bolstering the credibility of the analysis [135].

As the study progresses, we will provide willing participants with updates on how the research is being used to enhance accountability and trust. Data collection and analyses for objectives 2 and 3 will begin in April and end in December of 2026.

## Discussion

The proposed study employs a solutions-oriented, sequential mixed-methods design to explore the intersectional barriers faced by women academic position holders, particularly racialized women, in PC and PH research in Canada. The study is structured in two phases: a quantitative phase to collect and analyze demographic data, followed by a qualitative phase involving in-depth interviews. This design allows for a comprehensive understanding of both the breadth and depth of the challenges faced by women academic position holders, facilitating the identification of effective strategies to promote equity and inclusion. While there is existing research on gender and racial disparities in academia (science and medical specialty disciplines), our comprehensive review of the literature indicates there are few studies that examine the role of race *and* gender across career stages in PC and PH in higher education, and there are no studies from Canada. As such, this gap underscores the need to investigate the intersectional challenges faced by diverse PC and PH researchers, facilitating the identification of interventions for promoting gender and racial equity in research. The study’s recommendations can include equitable hiring, faculty support, and leadership advancements.

### Rigour

The sequential mixed-methods design combines the strengths of both quantitative and qualitative approaches, providing a comprehensive understanding of the research problem. This methodological triangulation enhances the credibility of the findings by corroborating data from different sources [136]. Moreover, employing maximum variation sampling will ensure a diverse representation of participants across different career stages, ethnicities, and academic backgrounds. This approach enhances the transferability of the findings to a broader context [137]. Confirmability will be achieved through reflexivity, where researchers will continuously reflect on their own biases and assumptions and how these may influence the research process and findings [137].

### Knowledge Dissemination

The knowledge dissemination plan for this study aims to reach a broad range of stakeholders through various channels to maximize its impact. Findings will be shared with the academic community via peer-reviewed journal articles and conference presentations, targeting academic and leadership position holders to stimulate discussions on equity and systemic change within institutions. Policy briefs and reports summarizing key findings and recommendations will be distributed to university administrators, deans, human resources departments, equity-focused offices, and funders to inform the development of more inclusive hiring practices, faculty support programs, and leadership initiatives. Additionally, workshops and webinars will engage professional associations and advocacy groups to promote widespread acceptance of strategies for change. Outreach to racialized women will ensure that underrepresented groups are represented and that their voices are included in conversations about academic equity. Lastly, key findings and resources will be shared on social media platforms, university websites, and open-access forums to engage students, early career and marginalized academic position holders, fostering broader awareness and ongoing dialogue on equity and inclusion in research.

### Study Limitations

Despite the comprehensive design of the proposed study, several limitations need to be acknowledged. Firstly, the scope is geographically limited to ON and BC, Canada, which may restrict the generalizability and transferability of the findings to other Canadian jurisdictions or international contexts [138], as the demographic and institutional characteristics of PC and PH academic position holders in other regions may vary. Secondly, if data on demographics are not available, the reliance on self-reported data for the quantitative phase may introduce response bias. Participants may under-report or over-report their experiences and demographic details, influenced by social desirability or recall bias [139]. Thirdly, institutional policies and practices regarding data sharing and transparency can affect the accuracy and comprehensiveness of the demographic and career-related data collected. Fourth, the qualitative phase relies on maximum variation sampling to ensure diverse perspectives [124], but the small sample size may limit the breadth of experiences captured. Despite best efforts, the focus on women and racialized women may overlook other intersecting identities, such as disability and sexual orientation, which may also cause unique barriers in academia [140–142].

## Conclusion

This study protocol outlines a comprehensive approach to examining the intersectional barriers faced by women, particularly racialized women, in Canadian PC and PH research. Utilizing critical race and intersectionality theories, the study aims to identify faculty characteristics, explore intersectional experiences, and recommend strategies to promote EDI in academic settings. The proposed study’s mixed-methods design, encompassing both quantitative demographic analysis and qualitative in-depth interviews, seeks to provide a holistic understanding of the systemic challenges and opportunities for women academic position holders at various career stages. The findings are expected to illuminate disparities in research productivity, institutional support, and workplace culture, contributing to a nuanced understanding of how gender, race, and other identities intersect and impact career trajectories in PC and PH academia. Despite the anticipated contributions, the study acknowledges limitations such as geographic restriction to ON and BC, potential response biases, and the challenges of capturing the full spectrum of intersectional identities and experiences. Ultimately, this study aims to inform the development of targeted interventions and policies that will foster a more equitable and supportive environment for women and racialized academic position holders in PC and PH. By addressing the identified barriers, the study seeks to enhance research productivity, career satisfaction, and retention of diverse talents in PC and PH, thereby contributing to more inclusive and innovative research that benefits a wider range of patients and society.

## Supporting information

S1 File Questionnaire. (PDF)

S2 File Interview Guide – DEI Stakeholders. (PDF)
