# Supplementary material for: Breaking barriers: A study protocol on unveiling gender, racial and other intersectional dynamics in post-secondary institutions and identifying solutions for advancing primary care and public health research
Source: PLoS One. 2026 Mar 17;21(3):e0344467. doi: 10.1371/journal.pone.0344467 (PMC12994812; doi:10.1371/journal.pone.0344467)
Supplement: S1 File — (PDF) [file pone.0344467.s001.pdf]

UNIVERSITY OF TORONTO
DALLA LANA SCHOOL OF PUBLIC HEALTH

# Survey

## Section 1: Demographic information

*This section collects basic demographic data to understand the diversity of respondents. All questions are optional. Please skip any questions that do not apply.*

1. What is your birth year?

   [Year]

2. What is your gender identity?
   - Genderqueer: Individuals who do not follow gender stereotypes based on the sex they were assigned at birth. They may identify and express themselves as "feminine men" or "masculine women" or as androgynous, outside of the categories "boy/man" and "girl/woman." People who are genderqueer may or may not identify as trans.
   - Genderfluid: Gender fluidity conveys a wider, more flexible range of gender expression, with interests and behaviours that may change from day to day. Genderfluid people do not feel confined by restrictive boundaries of stereotypical expectations of women or men. In other words, they may feel they are a woman some days and a man on others, or possibly feel that neither term describes them accurately.
   - Man / male: A person whose gender identity may or may not correspond with social expectations associated with being a man, masculinity, and/or male identity. People who identify as men may or may not identify as trans.
   - Nonbinary: An umbrella term for gender identities that fall outside of the man-woman binary
   - Questioning: When a person is exploring their own gender identity or is unsure with regards to their gender identity
   - Trans man: A person who was assigned female at birth and identifies as a man
   - Trans woman: A person who was assigned male at birth and identifies as a woman
   - Two-Spirit: An umbrella term encompassing gender and sexual diversity in Indigenous communities. Two-Spirit people often serve integral and important roles in their communities, such as leaders and healers. There are

many understandings the term Two-Spirit – and this English term does not resonate for everyone. Two-Spirit is a cultural term reserved for those who identify as Indigenous.

- o Woman / female: A person whose gender identity may or may not correspond with social expectations associated with being a woman, femininity, and/or female identity. People who identify as women may or may not identify as trans.
- o Other (please specify): [Open text box]

3. What is your racial background? Select all that apply.
   - ☐ African
   - ☐ European
   - ☐ East Asian
   - ☐ South Asian
   - ☐ South East Asian
   - ☐ First Nations, Inuit, Métis, Indigenous (please specify): [Open text box]
   - ☐ Hispanic or Latina/Latino
   - ☐ Middle Eastern
   - ☐ Other (please specify): [Open text box]
   - ☐ Prefer not to answer

4. What is your current immigration status in Canada?
   - o Canadian citizen (born in Canada)
   - o Canadian citizen (naturalized)
   - o Permanent resident
   - o Temporary work permit holder
   - o International faculty on a visa
   - o Refugee status
   - o Other (please specify): [Open text box]

5. What is your sexual orientation? Select all that apply.
   - ☐ Asexual
   - ☐ Bisexual
   - ☐ Gay
   - ☐ Lesbian
   - ☐ Pansexual
   - ☐ Queer
   - ☐ Heterosexual/straight
   - ☐ Questioning/unsure
   - ☐ Two-Spirit
   - ☐ Other (please specify): [Open text box]

6. Do you identify as a person living with a disability or chronic health condition? Select all that apply.
   - ☐ Yes, physical/mobility disability (e.g., wheelchair user, chronic pain, musculoskeletal condition)
   - ☐ Yes, sensory disability (e.g., blindness/low vision, deafness/hard of hearing)
   - ☐ Yes, neurodivergence (e.g., autism, ADHD, dyslexia)
   - ☐ Yes, mental health condition (e.g., depression, anxiety, PTSD)
   - ☐ Yes, chronic health condition (e.g., diabetes, autoimmune disorder)
   - ☐ Yes, intellectual or development disability
   - ☐ Yes, other (please specify): [Open text box]
   - ☐ No

## Section 2: Professional and educational background

*This section explores your academic qualifications, career path, and clinical experience (if applicable).*

7. What are your educational qualifications and when were they obtained? Add as many as applicable.

| Degree | Year obtained |
|---|---|
| [Drop-down menu:<br>○ Bachelor's Degree (e.g., BA, BSc, BEd)<br>○ Master's Degree (e.g., MA, MSc, MEd, MSW)<br>○ Doctoral degree (e.g., PhD, EdD, DPhil)<br>○ Professional degree (e.g., MD, JD, DO, DDS, DVM, PharmD] | |
| As above | |
| As above | |

8. Have you taken any career breaks (e.g., parental, illness, or bereveament leave), and for how long? Add as many as applicable.

| Break | Year(s) taken |
|---|---|
| [Drop-down menu:<br>○ Parental<br>○ Illness<br>○ Bereavement<br>○ Other, please specify | |
| As above | |
| As above | |

9. What is your tenure status?
    - Tenure-track
    - Tenured
    - Continuing teaching stream (non-tenure)
    - Continuing research stream (non-tenure/research scientist)
    - Clinical faculty appointment (non-tenure track)
    - Contract-limited term (full-time)
    - Contract-limited term (part-time)
    - Casual contract

- ○ Sessional/Adjunct (course-by-course or short-term contract)
- ○ Ongoing continuing status (full-time)
- ○ Ongoing continuing status (part-time)
- ○ Soft-money position (primarily grant-funded)
- ○ Other (please specify): [Open text box]

10. What is your current academic position?
    - ○ Adjunct Faculty
    - ○ Assistant Professor
    - ○ Associate Professor
    - ○ Full Professor
    - ○ Other (please specify): [Open text box]

11. If you have a part-time status, what is your FTE status?

    [Slider. Increments: 0, 0.1, 0.2, ... 1.0]

12. What is/are your primary field(s) of research? Select up to three.
    - ☐ Environmental Health
    - ☐ Epidemiology
    - ☐ Global Health
    - ☐ Health Informatics
    - ☐ Health Policy
    - ☐ Health Services
    - ☐ Health Systems
    - ☐ Health Workforce
    - ☐ Primary Care
    - ☐ Public Health
    - ☐ Social Determinants of Health
    - ☐ Other (please specify): [Open text box]

13. Are you currently or have you previously been a practicing clinician?
    - ○ Yes, currently practicing
    - ○ Yes, but no longer practicing
    - ○ No, I have never been a clinician

    If yes, please briefly describe your clinical role(s)
    - Family Physician
    - Nurse/Nurse Practitioner
    - Occupational Therapist
    - Pharmacist
    - Physiotherapist
    - Psychologist
    - Social Worker

- Other: [Open text box]

# Section 3: Family and home life

*This section examines how caregiving responsibilities and family structures intersect with academic careers.*

14. What is your relationship status?
    - ○ Single
    - ○ Married
    - ○ In a domestic partnership
    - ○ Divorced
    - ○ Widowed
    - ○ Other (please specify): [Open text box]

15. If you have a spouse/partner, do they have a career or professional engagement?
    - ○ Yes, currently active
    - ○ Yes, but retired
    - ○ No

16. If you have children, please indicate how many, their type and birth year?

| Child number | Type | Birth year |
|---|---|---|
| | Drop-down menu:<br>○ Birth<br>○ Adopted<br>○ Fostered<br>○ Step-child<br>○ Etc. | |
| | | |
| | | |

17. Do you have other caregiving responsibilities (e.g., spouse/partner, elderly relatives, other dependents)?
    - ☐ Yes, daily
    - ☐ Yes, weekly/monthly
    - ☐ No

## Section 4: Institutional context

*This section asks about your current institution type, size, and funding structure to contextualize your experiences.*

18. Which of the following best describes your primary institution or professional environment?
    - Research university: Renowned for their research output and offer a wide range of undergraduate and graduate programs across various fields of study (*e.g., U15 Universities: University of Toronto , University of British Columbia, Dalhousie University, McGill University)*
    - Comprehensive university: Offer a mix of undergraduate, graduate, and professional programs, providing a broad-based education while also supporting research and innovation (*e.g., University of Waterloo, Simon Fraser University, Memorial University of Newfoundland, Université de Sherbrooke* )
    - Primarily undergraduate university: Focus mainly on undergraduate education and typically offer a small range of graduate programs (*e.g., Trent University, University of Northern British Columbia, Bishop's University, Mount Allison University* )
    - Community college, polytechnic, or CEGEP: Emphasis on applied education or vocational training (*e.g., Seneca Polytechnic, Southern Alberta Institute of Technology, Dawson College, Nova Scotia Community College*)
    - Research institute or think tank: Independent or affiliated organizations focused on primary care, public health, or policy research (*e.g., Wellesley Institute, Fraser Health Research Institute, Manitoba Centre for Health Policy, Institut National de Santé Publique du Québec*)
    - Teaching hospital or academic medical centre: Integrated clinical and research environment (*e.g., University Health Network, BC Children's Hospital, McGill University Health Centre, QEII Health Sciences Centre*)
    - Government agency or public health unit: Federal/provincial health departments or local public health units (*e.g., New Brunswick Department of Health, Ministére de la Santé et des Services Sociaux, Public Health Ontario, Alberta Health*)
    - Non-profit/NGO: Organizations focused on health advocacy or service delivery (*e.g., Canadian Public Health Association, Canadian Medical Association, Canadian College of Health Leaders*)
    - Other (please specify): [Open text box]

19. What was the date of your appointment to this institution?

    [Year]

## Section 5: Career Progression and Leadership

*This section investigates milestones like funding, publications, promotions, and leadership roles.*

20. How much total funding (in grants, fellowships, etc.) have you received **as a Principal Investigator** (PI) throughout your academic career?

    [Slider. Increments: $0, $100,000, $200,000 … $5,000,000+]

21. What sources of funding have contributed to your total funding? Rank order your top 3.
    - ☐ Canadian Institutes of Health Research (CIHR)
    - ☐ Social Sciences and Humanities Research Council of Canada (SSHRC)
    - ☐ Natural Sciences and Engineering Research Council of Canada (NSERC)
    - ☐ Other government funding (provincial, territorial or federal)
    - ☐ Charitable organizations (e.g Canadian Cancer Society, Heart & Stroke)
    - ☐ Private foundations or organizations
    - ☐ Corporate partnerships
    - ☐ University-based funding or internal grants
    - ☐ Other (please specify): [Open text box]

22. Have you been involved in any significant collaborative research projects or networks?

| Involvement | Project description | Funding Source | Year(s) [Start & Finish] |
|---|---|---|---|
| Drop-down menu:<br>○ Principal Investigator<br>○ Co-Principal Investigator<br>○ Co-Investigator<br>○ Collaborator<br>Etc. | [Open text box] | Drop-down menu:<br>○ CIHR<br>○ SSHRC<br>○ NSERC<br>○ Other | |
| | | | |
| | | | |

23. How many peer-reviewed publications have you authored or co-authored in total in the past 5 years?

- ○ 0-5 publications
- ○ 6-10 publications
- ○ 11-20 publications
- ○ 21+ publications

24. How many peer-reviewed publications have you published **as lead or senior author** in the past 5 years?
    - ○ 0-5 publications
    - ○ 6-10 publications
    - ○ 11-20 publications
    - • 21+ publications

25. Have you received any major academic awards or recognitions? Please select all that apply.
    - ☐ Research Chair or professorship (e.g., Canada Research Chair, Endowed Chair, Distinguished Professorship)
    - ☐ Major research award of prize (e.g., national or international scholarly awards, disciplinary prizes)
    - ☐ Career or lifetime achievement award (e.g., early-, mid-, or late-career recognition)
    - ☐ Teaching or educational excellence award (e.g., institutional, provincial, or national teaching awards)
    - ☐ Leadership or service recognition (e.g., academic leadership, service to profession or community)
    - ☐ Fellowship or academy membership (e.g., Royal Society of Canada, Canadian Academy of Health Sciences)
    - ☐ Honorary degree or honorary title
    - ☐ Other (please specify): [Open text box]

26. Approximately how many individuals do you **currently** mentor in an academic or professional capacity?
    - ○ 0
    - ○ 1-2
    - ○ 3-5
    - ○ 6-10
    - ○ More than 10

27. Which of the following best describe the people you mentor? Select all that apply.
    - ☐ Undergraduate students
    - ☐ Graduate students
    - ☐ Clinical Trainees
    - ☐ Postdoctoral scholars
    - ☐ Early career researchers

☐ Other (please specify): [Open text box]

28. Are you currently or have you previously held formal leadership positions in academic or research settings?.

    *a)* Research leadership roles *(e.g., research director, program lead, scientific lead, prinicipal investigator of large research initiatives)*

    ☐ Yes, I actively sought leadership roles
    ☐ Yes, I was recruited/invited into leadership
    ☐ No, but I was/am interested in leadership
    ☐ No, because I am not interested in research leadership

    *b)* Administrative leadership roles *(e.g., department chair, division head, associate dean, dean, senior faculty leadership roles)*

    ☐ Yes, I actively sought leadership roles
    ☐ Yes, I was recruited/invited into leadership
    ☐ No, but I was/am interested in leadership
    ☐ No, because I am not interested in academic leadership

29. For leadership roles you've held, what type of support did you receive? Select all that apply.

    ☐ Formal leadership training
    ☐ Mentorship from senior leaders
    ☐ Reduced teaching/service workload
    ☐ Clear succession planning
    ☐ None—assumed role without support
    ☐ Other (please specify): [Open text box]

30. If you have sought but not attained leadership positions, what barriers have you faced? Select all that apply.

    ☐ Lack to mentorship/sponsorship
    ☐ Exclusion from "inner circles"
    ☐ Caregiving/disability needs
    ☐ Institutional policies that penalize parents/caregivers
    ☐ Institutional policies that hinder the advancement of non-tenured faculty
    ☐ Other reasons (please specify): [Open text box]

## Section 6: Experiences of Professional Discrimination

*Please answer the following questions with respect to your* ***professional academic experiences in your organization****.*

| | |
|---|---|
| 31. During my professional research experience (select all that apply),<br>a. I have encountered **inadequate recognition of my work** related to my identity.<br>b. I have felt a **lack of support** for professional growth (*job opportunities, mentorship, recognition*) compared to my colleagues.<br>c. I have felt **excluded from academic collaborations** and/or networking opportunities.<br>d. I have felt **excluded from research collaborations** and/or networking opportunities by researchers in the field.<br>e. I have felt **invisible**.<br>f. I have experienced feelings of **isolation**.<br>g. I have been ignored or dismissed when asking questions or when asking for help.<br>h. I have felt that my department within the institution is **informally segregated**.<br>i. I have experienced **not being taken seriously.**<br>j. I have encountered **identity-based remarks** or comments.<br>k. I have encountered **identity-based discrimination** (*unfair or injurious distinction or treatment*).<br>l. I have had my academic ability and/or **intelligence minimized**.<br>m. I have been made to feel like **the way I speak is inferior**.<br>n. I have been treated with **less respect** than my colleagues.<br>o. I feel that people **treat me negatively**.<br>p. I have had **stereotypes** made about me.<br>q. I have experienced someone making **offensive jokes** about me or people like me.<br>r. I was unfairly **not hired** for a job.<br>s. I was unfairly **denied a position** after being offered a job.<br>t. I was unfairly **denied a promotion**.<br>u. I was unfairly **denied an award**.<br>v. I was unfairly **fired**. | 32. For each of the experiences of discrimination or unfair treatment you reported above, what do you believe was the **main factor contributing** to these experiences?<br>☐ Age<br>☐ Caregiving or family status<br>☐ Disability, chronic health condition, or neurodivergence<br>☐ Gender identity<br>☐ Immigration status or citizenship<br>☐ Indigenous Identity<br>☐ Language, accent, or manner of speaking<br>☐ Race/ethnicity<br>☐ Religion or spiritual beliefs<br>☐ Sexual orientation<br>☐ Skin colour or complexion<br>☐ Other (please specify): [Open text box] |

# Section 7: Institutional Support

This section addresses institutional supports across the different career stages: hiring, productivity, and promotion (mid-career).

33. How would you describe the current **productivity expectations** for faculty at your institution (e.g., grants, publications, teaching, service)?
    - Manageable—expectations align reasonably with time/resources
    - High but fair—challenging but achievable with support
    - Unsustainable—expectations exceed capacity without sacrifices
    - Unclear—no consistent standards communicated

34. Have **productivity expectations changed** since you began your position?
    - Yes, expectations have increased significantly
    - Yes, expectations have become more flexible
    - No meaningful change
    - I don't know

35. How **transparent** were promotion/tenure criteria at your institution?
    - Very transparent
    - Somewhat transparent
    - Unclear or inconsistent
    - Actively opaque
    - Not applicable

36. How accessible are leadership opportunities at your institution?
    - Very accessible—clear pathways exist
    - Somewhat accessible—but requires advocacy
    - Limited—informal "tap on the shoulder" system
    - Inaccessible—systemic barriers prevent advancement

37. Did you encounter any of the following **barriers** as a faculty member? Select all that apply.
    - ☐ Inequitable startup resource allocation
    - ☐ Inequitable service/teaching workloads
    - ☐ Inequitable opportunities to permit promotion
    - ☐ Lack of mentorship for advancement
    - ☐ Exclusion from collaborative networks

- ☐ Lack of opportunities to apply for grants, research chairs and professional development opportunities compared to colleagues
- ☐ Competitive environments in which colleagues and leaders attempt to take over your projects, grants or partnerships
- ☐ Caregiving/health needs conflicting with responsibilities
- ☐ No barriers

38. If you have taken **parental, medical,** or a **caregiving leave**, did you receive adequate institutional support?
    - Yes, with clear policies (e.g., tenure clock extension)
    - Somewhat, but support was inconsistent
    - No, faced professional penalties

If no, please describe your experience: [Open text box]

39. How **supported** do you feel overall within your current role at this institution?
    - o Very supported
    - o Somewhat supported
    - o Neutral
    - o Somewhat unsupported
    - o Very unsupported

40. If you have ever left academia (*other than for parental, medical, or a caregiving leave*), how long were you away?
    - o I have never left academia
    - o Less than 6 months
    - o 6-12 months
    - o 1-2 years
    - o More than 2 years

41. At any point in your career, have you seriously considered leaving academia?
    - o Yes, currently
    - o Yes, in the past
    - o No

## Section 8: Workplace Responsiveness

42. How **equitable** were hiring processes when you joined your current institution?
    - Very equitable
    - Somewhat equitable
    - Neutral
    - Somewhat inequitable
    - Very inequitable
    - Not applicable

43. How would you describe the **overall culture** within your department, school, or faculty?
    - Highly inclusive
    - Somewhat inclusive, but with some challenges
    - Neutral
    - Somewhat exclusionary
    - Highly exclusionary

44. In your experience, how effective are your institution's **diversity, equity, and inclusion (DEI) initiatives** in producing <u>meaningful change</u> (e.g., in hiring, promotion, workload, leadership)?
    - Very effective
    - Somewhat effective
    - Mixed or inconsistent
    - Somewhat ineffective
    - Very ineffective
    - Unsure/not enough information

45. How confident do you feel in your institution's ability to effectively **respond to formal reports of discrimination**?
    - Very confident
    - Somewhat confident
    - Neutral
    - Not very confident
    - Not at all confident

46. If you have raised concerns or feedback to institutional leadership (e.g., deans, chairs, HR), how is it **typically received**?
    - Actively addressed, with concrete action
    - Acknowledged but with little or no action taken
    - Minimized or dismissed

- I feared or experienced retaliation
- I have not raised concerns

**47.** What strategies would help improve fairness, opportunities, and overall experience for equity-deserving groups at your institution? : **[Open text box]**

48. Would you be interested in being contacted to participate in a follow-up interview for this study?
    - Yes [will open up another screen to capture email address]
    - No
