# Supplementary material for: Breaking barriers: A study protocol on unveiling gender, racial and other intersectional dynamics in post-secondary institutions and identifying solutions for advancing primary care and public health research
Source: PLoS One. 2026 Mar 17;21(3):e0344467. doi: 10.1371/journal.pone.0344467 (PMC12994812; doi:10.1371/journal.pone.0344467)
Supplement: S2 File — (PDF) [file pone.0344467.s002.pdf]

UNIVERSITY OF TORONTO
DALLA LANA SCHOOL OF PUBLIC HEALTH

Semi-structured Interview Guide – Faculty

Section 1: Entering academia

1. Let's begin by talking about your early career—when you were entering academia. Can you tell me the story of how you came to pursue a career in academia and research?
   Probes:
   - What early experiences or influences shaped your interest in research or teaching?
   - Were there people—such as family members, teachers, mentors, or community members—who encouraged or modeled this path?
   - Was academia something you always imagined for yourself, or did your path evolve over time? How?

2. Can you tell me about your experience with the hiring process when you first became faculty?
   Probes:
   - Did the process feel fair and transparent to you? What was the composition of the hiring committee?
   - Were there specific barriers you faced related to your identity or circumstances?
   - Did you apply for a targeted job opportunity for an equity-deserving population? To what extent do you think this is required to enter academia?
   - Were expectations clearly communicated, and did they feel equitable?
   - Were personal and family circumstances and responsibilities taken into account in any way?
   - Were there aspects of the process that you would have changed, based on your experience?
   - Were you offered fair compensation?

3. Thinking back to when you entered the academic workforce, what kinds of supports or obstacles did you encounter?
   Probes:
   - Were institutional policies or norms helpful or limiting at this stage?
   - Did mentorship or leaders play a role?

- Did you feel your identity (race, gender, etc.) influenced access to opportunities? Have there been moments when your overlapping identities created specific challenges—or opened up unexpected opportunities?
-

4. How did you experience the transition into faculty life? Was it what you expected?
   Probes:
   - How do you experience the culture within your faculty?
   - Were there any "unwritten rules" you had to learn?
   - Was the leadership supportive of you? Why or why not?
   - Did you feel there was differential treatment from leaders or colleagues compared to your peers based on your identity? If so, explain.

5. How do you define "research productivity" for yourself and your institution?
   - o What metrics or outcomes are most valued in your faculty (e.g., publications, grants, community impact)?
   - o How do these institutional definitions align or conflict with your personal goals or values as a researcher?
   - o Have you faced pressure to prioritize certain types of productivity over others? If so, how has this affected your work?
   - o Are there alternative forms of productivity (e.g., mentorship, public engagement) that you feel are undervalued?

6. What key factors influenced your research productivity?
   Probes:
   - How have institutional or departmental policies helped or hindered your research productivity?
   - Were there any pivotal moments where institutional support (or lack thereof) played a defining role in your academic progress?
   - How have your personal identity and background impacted your trajectory?
   - Have you experienced disparities in how responsibilities are assigned or opportunities distributed based on gender, race, or other aspects of identity?
   - Have you experienced instances of biased reviews as part of funding opportunities and journal reviews?
   - Have you experienced situations where colleagues or leaders have attempted to take over your opportunities or recognition for your work?

Section 2: Mid-career as faculty

7. Let's move now to your experiences once you were established as faculty. What has helped or hindered your career progression at this stage?
   Probes:
   - Have faculty-level policies or expectations shifted over time?

- Have you had access to equitable workloads, funding, or mentorship based on the responsibilities of your specific position?
- Have you had to make compromises in your research because of systemic constraints?

8. Can you describe any personal experiences or life events that affected your research productivity?
   Probes:
   - Are there particular constraints or advantages that affected the pace or direction of your academic progress?
   - Have you had to make career compromises due to personal responsibilities or identity-related barriers? If so, how did you navigate them?
   - Have your personal networks (family, friends, community) influenced your ability to navigate academia?

9. What has your experience been with institutional policies related to work-life balance?
   Probes:
   - What policies currently exist?
   - Have policies around parental leave, caregiving, illness, or flexibility worked for you?
   - Have there been gaps in support?

10. How have faculty-level practices shaped your access to grants, awards, or other forms of recognition?
    Probes:
    - Have you felt that your research is valued by funders or the institution?
    - Have you experienced biased reviews of your proposals or papers?
    - Have you experienced biases of your performance by funders or the institution?
    - Have you observed patterns in who receives visibility or acknowledgment?
    - How has funding affected your research productivity, agenda and practices?
    - Are there assumptions in academia about who belongs, what counts as valuable research, or how success is defined, that don’t reflect your experience?

11. Can you talk about how mentorship, collaboration, and professional networks have (or haven’t) supported your work?
    Probes:
    - Have you had access to mentorship? How has the quality of mentorship impacted your career progression?
    - Have power dynamics or identity factors affected your inclusion in partnerships or collaborations?

- How have networking opportunities influenced your career? How do your networking opportunities compare to those of colleagues or your expectations?
- How do you think invitations to present your research at lectures, keynotes, and so on shape your career, and how do your experiences in this regard compare to your colleagues' or expectations?

12. Can you describe your experience with promotion and/or tenure practices in your faculty?
    Probes:
    - Were the criteria and expectations for promotion or tenure clearly communicated?
    - Did the process feel equitable and transparent?
    - Were your contributions—especially to mentorship, equity, or service—properly recognized?
    - Did your identity influence how your work was assessed? Have you observed differences in how your peers' work is evaluated or rewarded?
    - Were your personal or family circumstances given due consideration and accommodation?
    - What would have made the process feel more fair or supportive?

13. Have you observed or experienced bias, harassment, violence or exclusion within your faculty? If so, how was it addressed?
    Probes:
    - How would you describe the overall climate toward equity-deserving faculty on campus?
    - Have you felt comfortable reporting incidents?
    - Were faculty leaders effective in addressing concerns?
    - Were there circumstances in which the leadership dismissed your concerns or instilled fear of retaliation to keep you silent?
    - What changes would improve institutional responses to these issues?

Section 3: Advancing to leadership

14. Now let's talk about senior roles and leadership within academia. Have you pursued or held leadership positions, like department chair, research lead, or senior faculty? What was the experience like?
    Probes:
    - Was it something you actively pursued or were invited to?
    - How accessible did leadership opportunities feel to you?
    - Were there mentorship or succession pathways?

15. If you are in a leadership role, what kind of support or preparation did you receive?

16. Do you feel leadership opportunities are distributed fairly within your faculty?
    Probes:
    - What factors serve as barriers or facilitators to obtaining leadership positions?
    - Are certain types of faculty more likely to be “tapped” for leadership?
    - Do you notice patterns in who holds decision-making power?
    - Have you seen tokenism or symbolic inclusion without real influence?

17. Have you ever experienced barriers to being seen as a leader or authority in your field or institution?
    Probes:
    - What does credibility or legitimacy look like in your academic setting?
    - Have you had to prove yourself in ways others may not have?

Section 4: DEI initiatives and participant priorities

18. Have you engaged with diversity, equity, and inclusion initiatives at your institution? What has that looked like?
    Probes:
    - Are DEI initiatives visible and well-resourced?
    - Do faculty actively participate in DEI initiatives?
    - What appears to be the overriding motive behind DEI initiatives?
    - Have you seen any tangible outcomes from these programs?
    - Who benefits most from DEI initiatives?

19. What policies, programs, or interventions do you believe would best support racialized women academics’ career progression and help create a more inclusive, equitable academic environment?
    Probes:
    - What policies or practices would make the biggest impact?
    - How do these policies or practices differ from those that currently exist?
    - How can faculty be better supported in research and leadership?
    - How can institutions improve faculty retention and mitigate burnout and attrition?
    - What structural or policy changes do you believe are necessary to address systemic racism and inequity in academia?
    - Are there successful models from elsewhere?

20. What are your own hopes, concerns, or priorities when it comes to equity in your field?
    Probes:

- Do you see changes over time in how racialized women are supported or excluded in academia? How does your experience compare to those before or after you?
- What would a more inclusive academic future look like to you?

Section 5: Closing reflections

21. Is there anything else you'd like to share about your experiences or suggestions for improving equity in primary care and public health academia?

22. Can you think of anyone else we should speak with to gain further insights on this topic?
